# Wastewater-based Epidemiology for COVID-19 Surveillance: A Survey

**Published:** 2024-03-22

**Authors:** Chen Chen, Gursharn Kaur, Aniruddha Adiga, Baltazar Espinoza, Srinivasan Venkatramanan, Andrew Warren, Bryan Lewis, Justin Crow, Rekha Singh, Alexandra Lorentz, Denise Toney, Madhav Marathe

**Affiliations:** a Biocomplexity Institute and Initiative, University of Virginia, Charlottesville, 22904, United States; b Virginia Department of Health, Richmond, 23219, United States; c Division of Consolidated Laboratory Services, Department of General Services, Richmond, 23219, United States; d Department of Computer Science, University of Virginia, Charlottesville, 22904, United States

**Keywords:** Wastewater, Epidemiology, Infectious disease, COVID-19, Survey

## Abstract

The pandemic of COVID-19 has imposed tremendous pressure on public health systems and social economic ecosystems over the past years. To alleviate its social impact, it is important to proactively track the prevalence of COVID-19 within communities. The traditional way to estimate the disease prevalence is to estimate from reported clinical test data or surveys. However, the coverage of clinical tests is often limited and the tests can be labor-intensive, requires reliable and timely results, and consistent diagnostic and reporting criteria. Recent studies revealed that patients who are diagnosed with COVID-19 often undergo fecal shedding of SARS-CoV-2 virus into wastewater, which makes wastewater-based epidemiology (WBE) for COVID-19 surveillance a promising approach to complement traditional clinical testing. In this paper, we survey the existing literature regarding WBE for COVID-19 surveillance and summarize the current advances in the area. Specifically, we have covered the key aspects of wastewater sampling, sample testing, and presented a comprehensive and organized summary of wastewater data analytical methods. Finally, we provide the open challenges on current wastewater-based COVID-19 surveillance studies, aiming to encourage new ideas to advance the development of effective wastewater-based surveillance systems for general infectious diseases.

## 1. Introduction

The pandemic of COVID-19 has posed significant challenges to public health systems and the global economy, thereby urging the need for effective surveillance methods to monitor the prevalence of the disease within communities. Conventional surveillance methods are heavily dependent on clinical test data, such as positive test cases and hospitalizations. The inherent limitation of clinical data-based surveillance methods lies in their limited coverage, labor intensity, and data staleness due to prolonged test procedures. In order to estimate the prevalence of the disease and detect potential outbreaks in a more timely fashion, wastewater-based epidemiology (WBE^1^) been identified as complementary to clinical methods.

**Figure 1:**
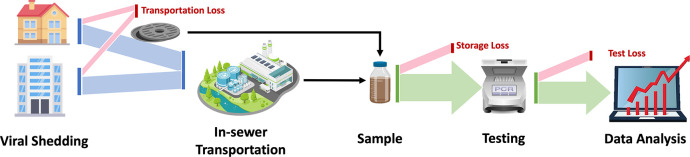
Overview of Wastewater-based Epidemiology Surveillance System.

WBE has been successfully used for monitoring the use of parmaceuticals Bischel et al. (2015), illicit drugs Zuccato et al. (2008), flu prevalence Heijnen and Medema (2011), and polio outbreaks Brouwer et al. (2018). Recent research suggests that monitoring the SARS-CoV-2 level in wastewater can be a reliable way to understand the disease prevalence in addition to the clinical test results Safford et al. (2022). Specifically, the wastewater samples can be collected from manholes in the targeted communities or from the wastewater treatment plants (WWTPs) in the sewersheds. The collected samples are then tested to quantify the concentration and the total load of the SARS-CoV-2 virus. The resulting viral concentration/load can be viewed as a comprehensive snapshot of disease prevalence within the community. By collectively analyzing the viral data from multiple timestamps, the trajectory of the disease may be estimated, which can be further used for trend projection. Figure 1 shows the overview of the wastewater-based epidemic surveillance system.

While a promising tool, wastewater-based COVID-19 surveillance is subject to some key limitations and challenges. The first challenge is the variability in viral shedding rates. Specifically, individuals of different symptom severity and age groups may contribute virus to the sewage system at significantly different rates, thus making it hard to approximate the infected population from wastewater viral load. Second, the wastewater viral load may get underestimated due to dilution in the sewer system, in-sewer transportation loss, degradation of the virus, and also the test procedures used. Such loss is inevitable and could lead to missed cases or delayed alerts for outbreaks. On the other hand, the sewershed population, wastewater flow variations, and sample methods may also affect the representativeness of the viral level in the test sample to the disease prevalence of the entire community. Therefore, approximating the actual viral load that flows into the sewage system from degraded signals requires careful modeling and analysis. The last challenge is the integration of wastewater analysis with conventional surveillance results (e.g. reported cases, hospitalization). Wastewater-based surveillance data provides a comprehensive snapshot of disease prevalence within the whole community but with potentially considerable degradation. In contrast, conventional surveillance results are accurate but only cover a limited portion of the infected population. Effectively combining the two data sources can be problematic as the studied populations are not well aligned.

In this paper, we survey the current literature that encompasses critical facets of wastewater-based surveillance for COVID-19, including wastewater sampling techniques, sample testing methodologies, data analysis methods, and available datasets at the global level. Furthermore, we highlight the ongoing challenges in the wastewater-based COVID-19 surveillance systems and hope to inspire continued innovation and development in the domain. It is worth mentioning that the data analytic methods for COVID-19 can be easily generalized to the surveillance tasks for other infectious diseases summarized in Kilaru et al. (2023).

### Differences with Existing Surveys.

Existing surveys on wastewater-basedCOVID-19surveillancearepredominantly focused on sampling methods, virus detection and quantification, and surveillance system design Sharara et al. (2021); Polo et al. (2020); Shah et al. (2022); Hamouda et al. (2021). In Ciannella et al. (2023); Li et al. (2023c), the two surveys have covered the correlation analysis between viral concentration and clinical test results, but the studies are not comprehensive enough to cover all the critical aspects of the analysis (e.g., sample type, sample frequency, correlation metrics). To the best of our knowledge, this is the first survey that focuses on summarizing the state-of-the-art analytical methods used in wastewater-based COVID-19 surveillance.

### Survey Structure.

The remainder of this survey is organized as follows, Section 2 and Section 3 briefly introduce the current advances in wastewater sampling and sample testing. Section 4 covers different aspects of wastewater analytic methods. Section 5 provides a comprehensive list of wastewater datasets for SARS-CoV-2 surveillance. Section 6 discusses the current limitations and challenges of wastewater-based COVID-19 surveillance systems, and Section 7 concludes the survey.

## 2. Wastewater Sampling

Sampling is a critical step for wastewater-based COVID-19 surveillance, which defines the surveillance scope for the disease. In particular, sampling through the sewage can effectively monitor the viral level at a community level or building level; while sampling at the wastewater treatment plant can estimate the infection level at the sewershed level. In addition to the sample location, sample frequency, sample type, and sample method may also affect the effectiveness of disease surveillance and prevalence estimation. This section summarizes the key findings for the above three aspects of wastewater sampling.

### Sample Frequency.

WBE is an important tool in monitoring the prevalence of SARS-CoV-2 in the community. Depending on the goal of surveillance, sampling frequency can vary. To screen for the presence of the virus, sampling once per week may be sufficient. To identify infection trends, at least three sampling points within a trend period of interest are needed. The National Wastewater Surveillance System (NWSS) suggests using a 15-day surveillance window for trend reporting CDC.

### Sample Type.

Wastewater samples can be categorized into two different types: (1) untreated wastewater from upstream sewage networks like manholes or treatment plant influent, and (2) treated wastewater from primary sludge in the treatment plant after the first solids removal stage. The advantage of using untreated wastewater from the upstream network or influents is that it can reflect fine-grained viral levels in targeted communities Layton et al. (2022); Cohen et al. (2022); Rondeau et al. (2023). However, most untreated wastewater samples need to be concentrated prior to viral extraction. For the treated wastewater samples from primary sludge, the concentration step can be eliminated but the viral level in the sample can only be used to evaluate the disease prevalence in the entire sewershed.

### Sample Method.

To collect wastewater samples, there are two commonly used methods: grab and composite. The grab method collects a fixed amount of wastewater at a certain time, while the composite method collectively pools multiple grab samples over a certain period of time. Compared with composite samples, grab samples only represent the viral levels at a single moment, which may vary by sampling time, fluctuations in wastewater flow, and study locations. In Gerrity et al. (2021), a wastewater study in Southern Nevada showed that the SARS-CoV-2 concentration in the composite sample is 10× higher than the early-morning grab samples. In Augusto et al. (2022), a similar study was conducted to evaluate the variability of SARS-CoV-2 RNA concentration in grab and composite samples from both wastewater treatment plants and sewer manholes in Brazil. Their study showed no significant difference between the viral concentrations of the grab and composite samples. In particular, the concentrations of composite samples showed greater agreement with concentrations of grab samples collected between 8 a.m. to 10 a.m. The low variability between the two types of samples was also observed in a study at a wastewater treatment plant in Norfolk, Virginia Curtis et al. (2020). However, the variability may get amplified when calculating the daily viral load (viral load = viral concentration × daily influent flow) from the viral concentrations. Such findings suggest that grab samples are sufficient to characterize SARS-CoV-2 concentrations. To effectively quantify the total viral load from wastewater, composite samples should be used.

## 3. Sample Testing

Sample testing aims to estimate the viral concentration from the wastewater samples, which directly affects the usefulness of downstream data analytic models. Generally, the testing step includes sample pre-processing and virus detection/quantification. To account for the viral loss in the testing step, some lab control methods were introduced to the process. Recent studies suggest that the tested viral concentration should also be normalized with the population served by the sewer system. Correspondingly, different normalization methods were incorporated into the virus quantification model. In this section, we summarize the key advances in sample pre-processing, virus detection and quantification, lab control methods, and normalization methods.

### Sample Pre-processing.

The wastewater samples need to be properly processed before being tested. The purpose of sample pre-processing is to remove solids Jmii et al. (2021) and inactivate virus/bacteria Reynolds et al. (2022). To remove the solids from the sample, centrifugation, and filtration can be performed. Specifically, the filtration needs to be done with large pore sizes (5 *μm* or larger) per CDC’s guidance CDC. In Yanaç et al. (2022), the authors suggested that SARS-CoV-2 RNA might predominate in solids. Therefore, concentration methods focusing on both supernatant and solid fractions may perform better for virus recovery. For the viral inactivation, effective procedures include thermal treatment Calderón-Franco et al. (2022); McMinn et al. (2021), UV light Castiglioni et al. (2022); Pellegrinelli et al. (2022) or chemical treatment Tomasino et al. (2021). Another key step before sample testing is sample concentration, which can help with the detection of SARS-CoV-2 RNA. The concentration step is particularly helpful for untreated wastewater samples as compared to the treated samples as mentioned in the previous section. Effective concentration approaches include ultrafiltration Dumke et al. (2021); Hasing et al. (2021), filtration through electronegative membrane Barril et al. (2021); Jmii et al. (2021), centrifugal ultrafiltration Anderson-Coughlin et al. (2021), ultracentrifugation Zheng et al. (2022), polyethylene glycol (PEG) precipitation Alexander et al. (2020); Farkas et al. (2021), skim milk flocculation Pino et al. (2021); Philo et al. (2021), and aluminum flocculation Pino et al. (2021); Salvo et al. (2021).

### Virus Detection and Quantification.

With proper pre-processing and concentration, the wastewater sample is then ready to be tested for SARS-CoV-2 RNA detection and quantification. The key step for the method is to quantify the targeted genetic materials (i.e., SARS-CoV-2 N1, N2 and E genes Lu et al. (2020); Corman et al. (2020)) with the polymerase chain reaction (PCR). The main step for the PCR test is using special chemicals and enzymes to amplify the targeted genetic materials in cycles. Once the target genes are amplified, they become detectable by lab methods and can be further interpreted to get the viral concentration in the sample. The most common way for RNA detection and quantification is polymerase chain reaction (PCR)-based quantification Ni et al. (2021). In practice, there are different PCR procedures used for SARS-CoV-2 RNA quantification, including RT-LAMP (reverse transcription loop-mediated isothermal amplification) Amoah et al. (2021), RT-qPCR (reverse transcription-quantitative polymerase chain reaction) Ahmed et al. (2020a), variations of RT-qPCR La Rosa et al. (2020); Navarro et al. (2021), and RT-ddPCR (RT-droplet digital PCR) Flood et al. (2021). In addition to the viral concentration, the number of amplification cycles used to detect the target genes (i.e., the $C_{t}$ value) can also be used as a criterion to quantify the viral load. Specifically, the lower the $C_{t}$ value, the greater the amount of viral RNA present in the original sample and vice versa.

### Lab Control Methods.

The amount of SARS-CoV-2 virus in the wastewater sample is subject to loss during the sample pre-processing and testing steps. The lost amount may vary by sample quality and testing methods. To assess the lost amount during the process, a frequently used method is using matrix recovery control. A matrix recovery control is a virus that is biologically similar to SARS-CoV-2. Some commonly used control viruses include murine coronavirus (also called murine hepatitis virus), bacteriophage phi6, Pepper Mild Mottle virus (PMMoV), bovine coronavirus, bovine respiratory syncytial virus, and human coronavirus OC43 Ahmed et al. (2020b); Torii et al. (2022); Hata et al. (2020); LaTurner et al. (2021); Nagarkar et al. (2022). Specifically, the matrix recovery control is spiked into the wastewater sample at a known concentration prior to the pre-processing step. The concentration of the control virus will be tested again after the testing step. The ratio of the virus concentrations before pre-processing and after testing can be used to estimate the recovery rate of the SARS-CoV-2 virus during the entire procedure.

### Normalization.

To enable the comparison of viral concentrations across locations and over time, the raw concentrations often need to be normalized by the daily wastewater flow and the population served by the sewer system. As the number of people contributing to the sewershed may vary over time due to factors like tourism and commuting, it is critical to utilize human fecal normalization to account for such changes. Human fecal normalization aims to estimate the human fecal content by targeting the organisms that are specific to human feces. Commonly used fecal indicator viral molecular targets include Pepper Mild Mottle virus (PMMoV) and crAssphage Rosario et al. (2009); Wilder et al. (2021). In D’Aoust et al. (2021b), it was shown that PMMoV RNA is relatively stable under different environmental conditions and therefore can boost the correlation between viral signals and COVID-19 cases. The bacterial molecular targets include Bacteroides HF183 and Lachnospiraceae Lachno3 Seurinck et al. (2005); Feng et al. (2018).

## 4. Data Analytics for wastewater-based COVID-19 surveillance

**Figure 2:**
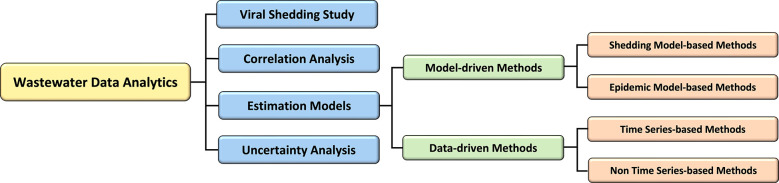
The overview of wastewater data analytics.

In this section, we review the current literature on wastewater data analytic methods from four perspectives, which include viral shedding studies, correlation analysis, estimation models, and uncertainty analysis. Specifically for the estimation models, we divide the current methods into model-driven methods and data-driven methods. The organization of this section is illustrated in Figure 2.

### 4.1. Viral Shedding Studies

The existing viral shedding studies are focused on quantifying the amount of SARS-CoV-2 virus in different types of human waste from infected individuals and the shedding duration of the virus.

#### Shedding Amount.

Gupta et al. (2020) reviewed the literature describing COVID-19 patients tested for fecal virus. The review shows that only 53.9% of the infected individuals tested for fecal RNA were positive. A more detailed study was conducted in Jones et al. (2020), which suggests that the SARS-CoV-2 RNA can be detected not only in feces but also occasionally in urine. The likelihood of SARS-CoV-2 being transmitted via feces or urine appears much lower due to the lower relative amounts of virus present in feces/urine. Consequently, the likelihood of infection due to contact with sewage-contaminated water (e.g. swimming, surfing, angling) or food (e.g. salads, shellfish) is extremely low or negligible based on very low abundances and limited environmental survival of SARS-CoV-2. Similar findings were also discovered in Wölfel et al. (2020), where a virological assessment of hospitalized patients with COVID-19 was conducted. Their study indicates that the infectious SARS-CoV-2 virus is exclusively derived from throat or lung samples, but never from blood, urine, or stool samples.

To calibrate the shedding rate of infected individuals, Schmitz et al. studied the WBE for SARS-CoV-2 by enumerating the asymptomatic COVID-19 cases in a university campus Schmitz et al. (2021). The study found that 79.2% of SARS-CoV-2 infections were asymptomatic and only 20.8% were symptomatic. To calculate the shedding rate, positive detected cases from the day before, day-of, and four days after sampling were included in the count of infected individuals contributing to viral shedding. The results showed that the mean fecal shedding rate by the N1 gene was 7.30 ± 0.67 log_10_ gc/g-feces (log gene copies per gram-feces).

In addition to the general shedding study on infected individuals, a later study was conducted to explore the association between patient ages and viral shedding amount based on the data from two wastewater sites in Massachusetts Omori et al. (2021). Specifically, the viral load in wastewater was modeled as a combination of viruses contributed by different age groups. By incorporating the case count delay, the wastewater viral load was fitted with the daily case count by different age groups. The results indicate that the virus contribution rate of patients from the 80+ yr age group can be 1.5 times larger than the corresponding rate of patients from the 0–19 yr age group.

#### Shedding Duration.

A study from Gupta et al. suggests that the duration of fecal viral shedding mostly ranges from 1 to 33 days after a negative nasopharyngeal swab Gupta et al. (2020). Similar findings were also reported in Wu et al. (2020). Moreover, Wölfel et al. (2020) reveals that fecal virus shedding peaks in the symptomatic period, and declines in the post-symptomatic phase. Miura et al. (2021) modeled the viral shedding kinetics with the collected data under the Bayesian framework. In particular, the duration of viral shedding and the concentration of virus copies in feces over time are jointly estimated. The results showed that the median concentration of SARS-CoV-2 in feces was 3.4(95% CrI^2^: 0.24–6.5) log gc/g-feces over the entire shedding period, and the duration of viral shedding is 26.0 days (95% CrI: 21.7–34.9) from symptom onset date.

### 4.2. Correlation Analysis

The correlations between the wastewater viral level and the clinical data (e.g. cases, hospitalization, death) are extensively studied in the current literature. Table 1 summarizes the correlation studies by their study location, sampling information (i.e., sampling site, method, frequency, and sampling period), and correlation details (i.e., correlation types, correlation variables, correlation strength, and time lag between the two variables). Specifically, the ‘Var. 2 lag’ column represents the lag of clinical data (i.e., *variable* 2) to viral levels (i.e., *variable* 1). Therefore, a negative lag time means the corresponding clinical data is leading the wastewater viral data. A positive lag time means the clinical data is lagging the viral data.

#### Correlation Metrics.

To evaluate the correlation between viral data and clinical data, some commonly used metrics include Pearson correlation, $R^{2}$ for the linear regression model, Spearman’s rank correlation, and Kendall’s τ correlation. Assume that the time series for wastewater viral data and clinical data are $X=\left\{x_{1},x_{2},\ldots ,x_{n}\right\}$ and $Y=\left\{y_{1},y_{2},\ldots ,y_{n}\right\}$ where the data pairs $\left(x_{t},y_{t}\right)$ are aligned at timestamp t. The correlations between the two time series under different metrics are defined as follows:

*Pearson correlation*: the Pearson correlation $r_{XY}$ between time series X and Y is defined as

(1) $$r_{XY}=\frac{\sum_{i=1}^{n}\;\;\left(x_{i}\;-\;\overset{‾}{x}\right)\left(y_{i}\;-\;\overset{‾}{y}\right)}{\sqrt{\sum_{i=1}^{n}\;\;{\left(x_{i}\;-\;\overset{‾}{x}\right)}^{2}}\sqrt{\sum_{i=1}^{n}\;\;{\left(y_{i}\;-\;\overset{‾}{y}\right)}^{2}}}$$

where $\overset{‾}{x}=\frac{1}{n}\sum_{i=1}^{n}\;x_{i}$ and $\overset{‾}{y}=\frac{1}{n}\sum_{i=1}^{n}\;y_{i}$ are the mean of the two series. The correlation score has a value between −1 and 1, which reflects the linear correlation of variables. One practical problem of Pearson correlation is its inability to handle noisy data. Specifically, when X and Y are both noisy, the correlation of the two series is not guaranteed to be low, which may lead to false positive correlation results.

$R^{2}$ *for linear regression model*: assume that the clinical data Y can be fitted by the wastewater viral data X with linear regression model (i.e., ${\overset{ˆ}{y}}_{i}=a+bx_{i}$ ), then the coefficient of determination $R^{2}$ can be calculated as

(2) $$R^{2}=1-\frac{\sum_{i=1}^{n}\;\;{\left(y_{i}\;-\;{\overset{ˆ}{y}}_{i}\right)}^{2}}{\sum_{i=1}^{n}\;\;{\left(y_{i}\;-\;\overset{‾}{y}\right)}^{2}}$$

The $R^{2}$ for the linear regression model can be used as a complementary metric for Pearson correlation as it can effectively rule out the correlation between noisy data.

##### Spearman’s rank correlation:

The Spearman’s rank correlation is used to evaluate the rank consistency between two data series. To calculate the correlation between X and Y, the two time series need to be converted into series of ranks $R_{X}$ and $R_{Y}$. The correlation coefficient would then be calculated as the Pearson correlation between $R_{X}$ and $R_{Y}$. The advantage of Spearman’s correlation is that X and Y can be related by any monotonic function rather than the linear correlation as in Pearson correlation.

##### Kendall’s τ correlation:

The Kendall’s τ correlation is defined by the concordance of data pairs. Specifically, for any pairs of data $\left(x_{i},y_{i}\right)$ and $\left(x_{j},y_{j}\right)$, the two pairs are considered concordant if the sort order of $\left(x_{i},x_{j}\right)$ and $\left(y_{i},y_{j}\right)$ agrees. Based on that, the correlation can be calculated as

(3) $$\tau =\frac{\text{\#concordant}\;\text{pairs}\;-\;\text{\#disconcordant}\;\text{pairs}}{\text{total}\;\text{pairs}}$$

The Kendall’s τ correlation is similar to Spearman’s rank small and when there are many tied values in the time series.

#### Influential Factors for Correlation.

The correlation strength between wastewater viral data and clinical data can be affected by many factors. Li et al. (2023c) conducted a systematic review and meta-analysis on the correlation between SARS-CoV-2 RNA concentration and COVID-19 cases. The review suggested that the correlation coefficients are potentially affected by environmental factors (e.g. temperature, humidity), epidemiological conditions (e.g. vaccination rate, clinical test coverage), WBE sampling design (e.g. sampling method and frequency), and catchment population (e.g. human mobility, demographics of inhabitants) Li et al. (2023c); Jiang et al. (2022); Rasero et al. (2022); Kuhn et al. (2022); Pillay et al. (2021). In particular, larger variations in air temperature and clinical testing coverage, and the increase of catchment size have strong negative impacts on the correlation between viral concentration and COVID-19 cases. The sampling techniques have a negligible impact on the correlation but increasing the sampling frequency can improve the correlation.

Moreover, extensive correlation studies suggested that the correlation between viral concentration and new cases (either daily new or weekly new cases) is stronger than that of active cases and cumulative cases. Also, as the shedding duration of the SARS-CoV-2 virus can be as long as several weeks, the correlations between wastewater viral data and reported cases are often stronger in the pre-peak phase than in the post-peak phase Róka et al. (2021). Normalizing viral data with fecal indicators can also improve the analysisRóka et al. (2021); Scott et al. (2021); Tandukar et al. (2022); Nagarkar et al. (2022); D’Aoust et al. (2021a,b); Perez-Zabaleta et al. (2023); Mohapatra et al. (2023). In addition to the aforementioned factors, the availability of home test kits has significantly affected the correlation between wastewater viral data and clinical data. Varkila et al. (2023) analyzed the time series of 268 counties in 22 states from January to September 2022. The study showed that SARS-CoV-2 wastewater metrics accurately reflected high clinical rates of disease in early 2022, but this association declined over time as home testing increased.

#### Varying Lag Time.

Aside from the correlation strength, many existing correlation studies also investigated the lag time between clinical data and wastewater viral load. The lag time may vary significantly by time, location, and catchment population due to the variant accessibility of testing resources and epidemiological conditions of the population Zhao et al. (2022); Kuhn et al. (2022); Bertels et al. (2023); Acosta et al. (2022); López-Peñalver et al. (2023); Belmonte-Lopes et al. (2023). In most cases, the viral load in wastewater is a leading indicator for clinical data, with leading time ranging from 1 day to 2 weeks during peak times considering the time-lag between infection and test confirmation, and asymptomatic infections Yanaç et al. (2022); Lemaitre et al. (2020). However, the viral load may become a lagging indicator during the infection declining phase due to prolonged viral shedding duration Gerrity et al. (2021). On the other hand, the lag time for different clinical data types may follow different distributions as well. In general, the lag times of positive tests are shorter than the hospitalization admissions. The lag of hospitalization is further shorter than the death cases Peccia et al. (2020); D’Aoust et al. (2021a); Krivoňáková et al. (2021).

#### Correlation with Estimated Prevalence.

In some areas, the wastewater viral data was used to estimate the disease prevalence in the sewersheds by leveraging the personal shedding rate and Monte Carlo simulations Wang et al. (2021); de Freitas Bueno et al. (2022). The estimated prevalence was found to be significantly higher than the reported clinical cases in the area due to asymptomatic cases and unreported cases. To thoroughly understand the gap between disease prevalence and reported cases, Layton et al. performed randomized door-to-door nasal swab sampling events in different Oregon communities to infer the community COVID-19 prevalence Layton et al. (2022). The estimated prevalence data was then compared with the reported positive cases and the wastewater concentration in the community. Statistical results show that the wastewater viral concentrations were more highly correlated with the estimated community prevalence than with clinically reported cases. Similar results were also observed in Claro et al. (2021); Pillay et al. (2021); González-Reyes et al. (2021); de Sousa et al. (2022); Saththasivam et al. (2021).

### 4.3. Estimation Models

#### 4.3.1. Model-driven Methods

##### Shedding Model-based Methods.

The key idea for shedding model-based estimation methods is to directly use viral concentration/load and human shedding profiles to estimate the total infected population. The pioneer work was proposed in Ahmed et al. (2020a, 2021), which studied the WBE for SARS-CoV-2 in Australia. In particular, the prevalence of SARS-CoV-2 in the sewershed was estimated using the following formula:

(4) $$I=\frac{c\;*\;f}{p\;*\;S}$$

with

(5) $$c=\frac{RNA\;copies}{liter\;wastewater}f=\frac{liter\;wastewater}{day}\;p=\frac{gram\;feces}{person\;per\;day}s=\frac{RNA\;copies}{gram\;feces}$$

where the infected population I is derived from the viral concentration c, wastewater flow rate f, fecal production rate p, and fecal shedding rate s. The uncertainty and the variability of the independent variables were approximated using a Monte Carlo approach, which yielded a reasonable result that agrees with the clinical observations. Similar analysis was also applied in Brazil Claro et al. (2021); de Sousa et al. (2022), South Africa Pillay et al. (2021), Mexico City González-Reyes et al. (2021), Tehran Amereh et al. (2022), Winnipeg Yanaç et al. (2022), Southern Nevada Gerrity et al. (2021), Denmark Nauta et al. (2023), and Qatar Saththasivam et al. (2021). The simple shedding model in Equation 4 can be easily modified to account for the viral decay Yanaç et al. (2022), the variability on shedding load, the infection-to-confirmation case delay Fernandez-Cassi et al. (2021), and urine viral shedding Pillay et al. (2021) scenarios. In de Sousa et al. (2022), the model is further developed into a user-friendly web application, pySewage de Sousa et al., to predict the number of infected people based on the detected viral load in wastewater samples, which may be applied to monitor ongoing outbreaks.

##### Epidemic Model-based Methods.

**Figure 3:**
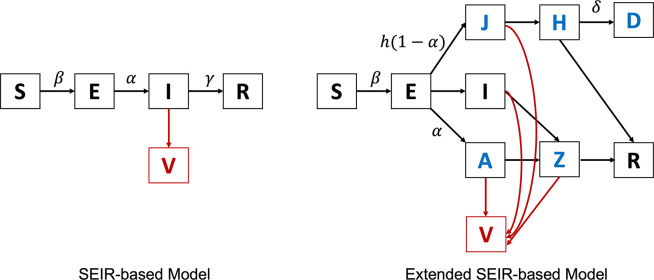
The SEIR model and extended SEIR model used in the current literature.

Another line of model-driven methods is to fit the wastewater data into epidemiological models like the susceptible-exposed-infectious-recovered model (SEIR model Anderson and May (1979)) to infer the dynamics of the disease. The framework was first proposed in McMahan et al. (2021). Specifically, the framework assumes that the spread of COVID-19 follows the SEIR model and that the viral load in wastewater is solely contributed by the infected population as illustrated in the left panel of Figure 3. Let $V_{ij}(t)$ denote the virus shed by individual i on day t, who become infected on day j, then $V_{ij}(t)$ can modeled by a simple equation below

(6) $$V_{ij}(t)=\delta_{ij}\{10^{\frac{\phi_{ij}(t-j)}{5}}I(j<t\leq 5+j)+10^{\psi_{ij}-\frac{\left(\phi_{ij}-\psi_{ij})(t-5-j\right)}{5}}I(t>5+j)\}$$

where $\delta_{ij}$ is the number of grams of feces contributed by the *i*th individual who was infected on the *j*th day, $\phi_{ij}$ is the log_10_ maximum RNA copies per gram of feces being shed, and $\psi_{ij}$ is the log_10_ RNA copies per gram of feces being shed 25 days after being infected. To further account for viral decay in the sewage system, a holding time and system temperature-dependent decay model is applied to $V_{ij}$ to approximate the viral loss in the collected samples. The proposed framework was fitted into the wastewater surveillance data in South Carolina from May 2020 to August 2020. The model prediction reveals that the rate of unreported COVID-19 cases was approximately 11 times than that of confirmed cases, which aligns well with the independent estimation of the ascertainment rate in South Carolina. Following the same framework, McMahan et al. propose to calibrate the SEIR model within a small community for fine-grained analysis McMahan et al. (2022). The study was carried out on a university campus by analyzing the viral RNA copy rates in sewage and the number of SARS-CoV-2 saliva-test-positive individuals among students McMahan et al. (2022). A strong correlation was observed between the RNA copy rates and the number of infected individuals. The study also suggested that the most sensitive parameter in calibrating the SEIR model is the maximum shedding rate. Regressing the saliva-test-positive infected individuals on predictions from the SEIR model based on the RNA copy rates yielded a slope of 0.87, which further demonstrated the effectiveness of the proposed framework. In Phan et al. (2023), Phan et al. extended the framework to incorporate the effect of temperature on viral loss into the model. The extended model was tested on the wastewater data in the Greater Boston Area from October 2020 to January 2021. The results showed that the model can successfully recapitulate the temporal dynamics of viral load in wastewater and predicted the true number of cases peaked earlier and higher than the number of reported cases by 6–16 days during the second wave of the pandemic in the area.

Directly inferring the SEIR model from the viral load in wastewater may yield unstable results due to noisy viral fluctuations. To address this issue, some statistical models were explored to reconstruct the epidemic model. Fazli et al. (2021) proposed to utilize the partially observed Markov processes model (POMP King et al. (2016)) to infer the population in *S*, *E*, *I*,*R* compartments respectively from the observed viral load and reported cases. Depending on the usage of observed data, three different variants were derived from the framework, which includes “SEIR-VY”, “SEIR-V” and “SEIR-Y”. Specifically, model “SEIR-VY” uses both viral load and case counts to fit the parameters, whereas model “SEIR-Y” and “SEIR-V” utilizes only case counts and viral load, respectively. The evaluation results demonstrated that a simple SEIR model based on viral load data can reliably predict the number of infections in the near future. Another direction of the study was to use the extended Kalman filter (EKF Kalman (1960)) to reconstruct the SEIR model Proverbio et al. (2022). The proposed framework was used to infer shedding populations, the effective reproduction number, and future epidemic projections. The framework was tested on the wastewater data from different regions. The results showed that the inferred case number is well correlated with the true detected case numbers with correlation coefficients ranging between 0.7 and 0.9. The study also validated that frequent sampling improves the model calibration and the subsequent reconstruction performance.

The limitation of the previously mentioned SEIR-based framework is that it assumes all the infected individuals follow the same shedding model. In reality, the shedding models of asymptomatic infections and hospitalized infections may vary significantly from each other. To address this issue, Nourbakhsh el al. presented an extended SEIR model as illustrated in the right panel of Figure 3 Nourbakhsh et al. (2022). Specifically, the infected individuals are further categorized into four subgroups: infection (I), infectious and later admitted to hospital (J), asymptomatic infectious (A), and hospitalized (H). Furthermore, considering some recovered cases may still shedding virus through feces, the recovered group is also divided into two subgroups: non-infectious but still shedding virus (Z) and recovered (R). The model was fitted by the clinical data (both hospitalization and confirmed cases) from three Canadian cities and has provided good estimation on actual prevalence, effective reproduction number, and future incidences. In addition, the model was also used to perform exploratory simulations to quantify the effect of surveillance effectiveness, public health interventions, and vaccination on the discordance between clinical and wastewater data.

The aforementioned frameworks are predominately based on single-strain epidemic analysis, which cannot effectively deal with the spread dynamics of multiple strains. In Pell et al. (2023), Pell et al. presented a four-dimensional modified SIR model to study disease dynamics when two strains are circulating in the population. The study was applied to understand the emergence of the SARS-CoV-2 Delta variant in the presence of the Alpha variant using the wastewater data from Massachusetts. In the model, a time delay is incorporated to account for temporary cross-immunity induced by the previous infection with the established (or dominant) strain. The study finds that the time delay does not influence the stability of equilibrium and is hence a harmless delay. However, the equilibrium is governed by the basic reproduction numbers of the two strains in nontrivial ways due to the inclusion of cross-immunity.

#### 4.3.2. Data-driven Methods

##### Time Series-based Methods.

In exploiting the predictive power of the wastewater data from a data-driven perspective, some time series-based methods have demonstrated their effectiveness in short-term forecast tasks. In Karthikeyan et al. (2021), Karthikeyan et al. experimented with the multivariate autoregressive integrated moving average (ARIMA) model to predict the number of new positive cases from the historical case data, wastewater data, and sample collection date in San Diego from July to October 2020. Specifically, the model was used for 1-week to 3-week advance case predictions. To evaluate the model, the Pearson correlation r between the observed cases and predicted cases and the Root Mean Squared Error (RMSE) of predicted cases were calculated. For the 1, 2, and 3-week advance forecast tasks, the correlation coefficient and RMSE were r=0.79,0.69, and 0.47 and RMSE=50,59, and 70, respectively.

In Cao and Francis (2021), a vector autoregression (VAR) model was utilized to predict new cases from historical cases and viral concentration in Indiana (PA) from April 2020 to February 2021. The Mean Average Percentage Error (*MAPE*) for 1–3 week case predictions were 11.85%, 8.97% and 21.57%, respectively. The study suggests that short time series can reliably predict cases 1-week ahead but are not adequate for predicting cases 3 weeks ahead. To improve the robustness of long-term prediction tasks, a longer time series is needed. Moreover, the paper also studied whether different representations of viral data would affect the prediction results. Their study shows that the log-scaled representation of viral concentration has the best interpretation ability of the data, while the original viral concentration has a stronger forecasting ability under the VAR model framework.

The ARIMA model and VAR model were systematically compared in a wastewater surveillance study in Detroit from September 2020 to August 2021 Zhao et al. (2022). The study showed that the autoregression model with seasonal patterns (SARIMA) and the VAR model are more effective in predicting COVID-19 incidence compared to the ARIMA model. Specifically, the correlation between VAR predicted cases and observed cases is around 0.95 to 0.96 for the 1-week advance forecast task. Similarly, the correlation for the SARIMA-model is around 0.94 to 0.95. While for the ARIMA model, the correlation is only around 0.4 to 0.67.

Another line of time series-based methods is derived from the spatiotemporal methods, which take both spatial information of sewersheds and temporal information of viral load into account in the estimation model. In Li et al. (2023a), Li et al. proposed a spatially continuous statistical model that quantifies the relationship between viral concentration and a collection of covariates including socio-demographics, land cover and virus-associated genomic characteristics at the sewersheds while accounting for spatial and temporal correlation. The model is used to predict the weekly viral concentration at the population-weighted centroid of the 32,844 Lower Super Output Areas (LSOAs) in England, then aggregate these LSOA predictions to the Lower Tier Local Authority level (LTLA). In addition, the model is also used to quantify the probability of change directions (decrease or increase) in viral concentration over short periods.

##### Non-time Series-based Methods.

A wide range of regression models have been applied to the wastewater data for case prediction due to the ease of implementation and explanability. The simplest regression model assumes that the number of cumulative cases at time t+τ is linearly related to the viral concentration at time t, and has demonstrated its effectiveness for short-term case prediction Joseph-Duran et al. (2022). In Li et al. (2023b), Li et al. applied the random forest model to predict COVID-19-induced weekly new hospitalizations in 159 counties across 45 states in the United States of America (USA). In particular, different models were established to predict three different hospitalization indicators: weekly new hospitalizations, census inpatient sum, census inpatient average. For each hospitalization indicator, a variety of features, such as Community Vulnerability Index Smittenaar et al. (2021), vaccination coverage, population size, weather, viral concentration, and wastewater temperature, were fed into the model. The study showed that the model can accurately predict the county-level weekly new admissions, allowing a preparation window of 1–4 weeks. In addition, it also suggests updating the training model periodically to ensure accuracy and transferability, with mean absolute error within 4–6 patients/100k population for upcoming weekly new hospitalization numbers. In Aberi et al. (2021), Aberi et al. compared eight different regression models for COVID-19 surveillance with the wastewater data from four treatment plants in Austria from May to December 2020. The tested models include Linear Regression (LR), Polynomial Regression (PL), k Nearest Neighbor (KNN), Multilayer Perceptron (MLP), Support Vector Regression (SVR), Generalized Additive Models (GAM), Decision Tree (DT) and Random Forest (RF). The study showed that simple models like PL and KNN outperform more complex models such as GAM, SVR, and MLP with slight differences. Similarly, Vallejo et al. applied linear regression, generalized additive model and locally estimated scatterplot smoothing model (LOESS) for COVID case prediction in Northwest Spain Vallejo et al. (2022). In addition to the wastewater data, some relevant atmospheric variables (e.g. rainfall, humidity, temperature) are also considered in the models. The results showed that the LOESS model yields the least prediction error with $R^{2}=0.88$. The $R^{2}$ for the linear and GAM model are 0.85 and 0.87, respectively. By changing the prediction period, the study found that the reliability of the model predictions could change by time due to different causes such as the change of SARS–CoV–2 variants. In Anneser et al. (2022), the linear and the GAM model were compared with Poisson model and Negative Binomial model to predict the cases from the wastewater data in the three New England regions. The models that fit the data best were linear, GAM, and Poisson model with very small differences on $R^{2}$ and *RMSE*. The same set of models were tested on the wastewater data in Oklahoma city from November 2020 to March 2021, with some sociodemographic factors (e.g. age, race and income) considered in the models Kuhn et al. (2022). The best results were obtained using a multivariate Poisson model. Consistent with the finding in Vallejo et al. (2022), the performance of the Poisson model varies by the time of study. Specifically, its accuracy decayed from 92%, during November 2020 until the end of January 2021, to 59% during February and March 2021. In Morvan et al. (2022), the shedding model in (4) and gradient boosted regression trees (GBRT) were combined to estimate the COVID prevalence in England with the wastewater data from 45 sewage sites. The estimated prevalence was within 1.1% of the estimates from representative prevalence survey Morvan et al. (2022). In Xiao et al. (2022), the changing dynamics between the reported cases and wastewater viral load were explicitly studied. Specifically, the clinical reported cases were modeled as the convolution between the scaled wastewater data and an unknown transfer function. It was hypothesized that the transfer function could be fit by a beta distribution. The model was fit into the wastewater surveillance data in the Boston area from March 2020 to May 2021. The results showed that the transfer function has a broad peak and long tail before mid August 2020, indicating that the process of infected individuals getting counted as cases has a broad distribution, with some individuals getting reported very quickly but others taking up to weeks. In this case, wastewater viral load can be used as an early indicator of disease dynamics before clinical test results come back positive. After mid August, the transfer function becomes more sharply peaked, indicating that wastewater and reported cases track each other closely. Consequently, wastewater viral load have less utility as an early warning signal as increased clinical testing capacity effectively captures new infections in a timely manner.

In addition to the aforementioned simple regression models, some deep learning-based models are also explored for the wastewater-based epidemic surveillance tasks Zhu et al. (2022); Jiang et al. (2022); Li et al. (2021a); Galani et al. (2022). Specifically, the artificial neural network model (ANN) and adaptive neuro fuzzy inference system (ANFIS) have proven effective in different studies for case prediction tasks when compared with linear models and random forest Li et al. (2021a). By incorporating the catchment information, weather, clinical testing coverage, and vaccination rate features into the ANN model, the effective reproduction rate can be estimated as studied in Jiang et al. (2022).

Aside from the effectiveness of learning models, the features used to feed the learning models may also have an impact on the prediction results. In Li et al. (2021a), the study indicated that the air and wastewater temperature played a critical role in the prevalence estimation by data-driven models. Also, normalizing and smoothing the wastewater data Aberi et al. (2021) or transforming the viral load into log scale Vallejo et al. (2022) can help in fitting the models as well. To better understand the spread of the disease and the effect of public health response, Xiao et al. proposed to monitor the ratio between wastewater viral load and clinical cases (WC ratio) and the time lag between wastewater and clinical reporting in addition to viral load alone Xiao et al. (2022). Specifically, when the WC ratio is high, it implies that the existing testing capacity has not kept pace with exponentially rising new cases, which nevertheless are detected in wastewater surveillance. Conversely, a low WC ratio indicates that clinical tests are capturing the majority of infections reflected in wastewater viral load. When this ratio is stable and low, it implies that the existing testing capacity is sufficient to assess the extent of new infections. The time lag, on the other hand, may reflect the accessibility of test facilities. In Kuhn et al. (2022), Kuhn et al. showed the lag was significantly lower for areas with a higher household income and a higher proportion of the population aged 65 or older, but higher for areas with a high proportion of Hispanic inhabitants.

### 4.4. Uncertainty Analysis

**Figure 4:**
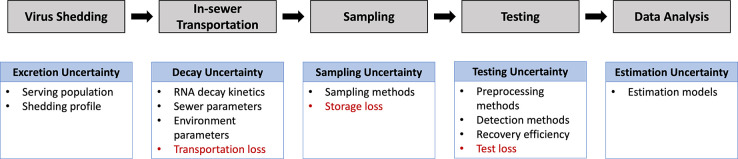
The uncertainty of wastewater-based COVID surveillance.

The accuracy of wastewater-based COVID-19 surveillance is limited by the uncertainty and inevitable viral loss introduced in each process step. Recall that the key steps for WBE systems are virus shedding, in-sewer transportation, sampling, testing and data analytics as shown in Figure 1, the uncertainties associated with those steps are illustrated in Figure 4.

In Li et al. (2021b), Li et al. systematically studied the uncertainty in estimating SARS-CoV-2 prevalence by WBE. The study suggested that the uncertainty caused by the excretion rate can become limited for the prevalence estimation when the number of infected persons in the catchment area is more than 10. As for the sampling methods, grab sampling contributed the highest uncertainty (around 30% on average) while a continuous flow-proportional sampling method showed <10%. The uncertainty introduced at the testing stage was the dominant factor. Therefore, it is important to use surrogate viruses as internal or external standards during the virus test process. Overall, WBE can be considered as a reliable complementary surveillance strategy for SARS-CoV-2 with reasonable uncertainty (20–40%).

## 5. Datasets

This section summarizes the global wastewater datasets that are publicly available in Table 2. For each dataset, the country, data granularity, area covered, time granularity, time span, current status, and corresponding website are listed. The data granularity represents the aggregation level of wastewater signals, which could range from building-level to country-level. The area covered column shows the monitoring area of the dataset. Time granularity is used to indicate the sampling frequency of the wastewater data. Specifically, for the datasets labeled with ‘>1/week’, more than one data point were observed in one week overall, but the actual weekly samples may vary along the course. The time span specifies the sampling period of the dataset, while the ‘live’ column indicates whether the data in the website is still getting updated or not. Lastly, the ‘website’ column gives the link to the dataset.

## 6. Challenges and Future Directions

Wastewater-based epidemiology has been used as an effective tool to complement conventional clinical testing methods for COVID-19 surveillance. Although substantial efforts have been made in the area, there are still many challenges to be addressed in future research. Three important problems that are worth exploring are identified as follows.

### Shedding Variability.

Current studies predominantly assume that the infected individuals follow a uniform shedding model with only a few works to account for the variability of the shedding profile. In fact, the shedding amount and duration of SARS-CoV-2 in feces can vary widely between individuals and over time. Factors such as the stage of infection, disease severity, vaccination condition, and individual health condition may all affect the shedding profile. As the shedding model is often directly used to estimate the disease prevalence together with the total viral load in the wastewater, it is therefore crucial to construct customized shedding profiles for different infected individuals.

### Sample Testing and Virus Quantification.

Long-term wastewater-based COVID-19 surveillance is an economical way to detect the outbreak of disease and emerging variants Karthikeyan et al. (2022); Lamba et al. (2022). One critical problem for the surveillance systems is the allocation of test resources. To be specific, given a limited budget for sample test resources, it is important to choose the sampling locations and frequency by considering the catchment size, and serving population in the area so that potential outbreaks can be detected as early as possible. On the other hand, the virus quantified in the wastewater sample may not reflect the actual amount of virus entering the sewage system because of the limited sensitivity of lab methods and viral decay in the sewage system. Therefore, it is essential to improve the lab testing methods and understand the virus decay model under various environmental parameters (e.g., temperature, wastewater pH, etc.).

### Data Analytics.

The majority of the existing literature takes the wastewater data as a standalone signal for epidemic analysis from site to site, while little effort has been made to study the wastewater data from multiple sites collectively for spatial-temporal pattern analysis. Compared to the standalone analysis, the spatial-temporal analysis is more useful for reconstructing the epidemic process at a panoramic scale. In particular, for some large metropolitan areas that can be divided into multiple sewersheds, local residents may contribute to different sites due to the commute from residential areas to commercial areas. In this way, it is hard to recover the disease spread process without tackling the interdependency across sites. The main obstacle to this research direction is the comparability of the data from different sites. Specifically, the sample collection methods, testing methods, and sewage system structure may vary by site. Correspondingly, the same viral load from different sewersheds may represent different epidemic conditions in reality. To this end, how to effectively compile those data into a uniform framework can be a challenging task to address. On the other hand, it is also critical to integrate the mobility pattern of local residents into the analytical model so that the dependency across the sites can be effectively unraveled.

Moreover, the uncertainties introduced in the wastewater analytic pipeline are not negligible as explained in Section 4.4. Therefore, it is important to quantify the uncertainty together with the prediction results to ensure the reliability of the results.

## 7. Conclusion

Wastewater-based epidemiology has been demonstrated as a powerful tool for COVID-19 surveillance and trend projection within communities. This survey summarizes the wastewater sampling techniques, sample testing methods, data analytical models, and the existing wastewater datasets at a global level. In particular, this survey provides a new taxonomy of data analytical models to help the researcher and practitioner form a systematic view of the area. Most importantly, the reviewed data analytical models can be easily generalized to many other infectious diseases, which can be referred to as guidance to build general disease surveillance systems. Moreover, the comprehensive wastewater datasets at different granularity can serve as a benchmark for validating new surveillance models at various scales. Last but not least, the challenges in the area are discussed, which may help inspire researchers in their future research directions.

## Figures and Tables

**Table 1:** Summarization of Correlation Studies between Viral Data and Clinical Data.

Location	Sampling				Correlation					Ref.

	Site/Pop.	Sample methods/Freq.	Study Period	Total Samples	Corr. Type	Corr. Var. 1	Corr Var. 2	Corr. Strength	Var. 2 Lag	

Sendai Japan	1 WWTP 360,000	Grab 2/week (Tue & Thu, 10am)	Aug 2020 - Feb 2021	51	Spearman	2 week positive sample percentage	2 week cumulative cases	0.4996 (*p* < 0.05)	9 days	Zhu et al. (2022)
						4 week positive sample percentage	4 week cumulative cases	0.7598 (*p* < 0.05)		

Detroit USA	3 WWTPs 492,000–1,482000	24h composite weekly	Aug 2021 - Feb 2022	407	Pearson	Sum of viral conc. from 3 WWTPs by N1 gene	7-day moving avg. of COVID-19 cases	0.62	5 weeks	Zhao et al. (2022)
						Sum of viral conc. from 3 WWTPs by N2 gene		0.64		

San Diego USA	1 WWTP 2.3 million	24h composite daily	Jul 2020 - Oct 2020	90	Pearson	Viral conc.	Daily hospital cases	0.75		Karthikeyan et al. (2021)

Budapest Hungary	3 WWTPs 1.8 million	24h composite & grab weekly (8 am-10 am)	Jun 2020 - Nov 2020	65	*R*^2^ of linear regression	Sum of viral load from 3 WWTPs	7-day rolling avg. active cases	0.589 (*p* < 0.001)	1 week	Róka et al. (2021)
							Daily new cases	0.67 (*p* < 0.001)	0	
							Hospital cases	0.36 (*p* < 0.01)	1 week	
							Death cases	0.235 (*p* < 0.05)	1 week	

Netherland	7 WWTPs 234,500 – 980,000	24h composite 8 days	Feb 2020 - Mar 2020	30	*R*^2^ of linear regression	log_10_ (viral load) by N1 gene	log 10 (reported cases)	0.66 (*p* < 0.01)		Medema et al. (2020)
						log_10_ (viral load) by N2 gene		0.59 (*p* < 0.05)		
						log_10_ (viral load) by N3 gene		0.79 (*p* < 0.001)		
						*C_t_*		0.77 (*p* < 0.001)		

Seville Spain	8 WWTPs 10,719 – 49,124	Grab weekly (9am-11am)	July 2020 - Jan 2021	199	Pearson	log_10_ (population normalized viral load)	Active cases	0.52	4 days	Rasero et al. (2022)
								0.51	6 days	

Oklahoma City USA	13 manholes 3,729 – 52,323	Grab 2/week (10am-1pm)	Nov 2020 - Mar 2021		*R*^2^ of linear regression	Avg. viral conc. from all locations	Reported cases	0.87 (*p* < 0.01)	7 days	Kuhn et al. (2022)
						Viral conc.	Reported cases in sewershed	0.41 – 0.95	4–10 days	

Los Angeles USA	5 WWTPs 150,000 – 4 million	24h composite weekly (1WWTP 2/week)	May 2020 - Mar 2021	250	Pearson	Viral conc. by N1 gene	New cases	0.88 (*p* ≪ 0.01)		Wang etal. (2021)
						Viral conc. by N2 gene		0.88 (*p* ≪ 0.01)		
						Smoothed viral conc. by N1 gene		0.94 (*p* ≪ 0.01)		
						Smoothed viral conc. by N2 gene		0.94 (*p* ≪ 0.01)		
	1 WWTP	24h composite daily	Aug 16–22 2020	~7		Smoothed viral conc. by N1 gene		0.96 (*p* < 0.005)	5 days	
						Smoothed viral conc. by N2 gene		0.96 (*p* < 0.005)	5 days	

Brazil	16 WWTPs & manholes 1,555,626 – 3,094,325	24h composite at WWTP 4h semi-composite at manhole	Jan 2021 - Jan2022		Spearman	Viral conc.	7-day cumulative new cases	0.41 – 0.63 (*p* < 0.005)		de Freitas Bueno et al. (2022)

Tulan University USA	Manholes <14,062	Grab weekly 10:30 am-11:30 am	Aug 2020 - Dec 2020	117	Spearman	Viral conc. by N1 gene	Reported cases	0.5067 (*p* < 0.0001)		Scott et al. (2021)
						Viral conc. by N2 gene	Reported cases	0.479 (*p* < 0.0001)		

Durham Canada	2 WWTPs 135,556 – 170,071	24h composite	Oct 2020 - Apr 2021	115	Pearson	Daily flow normalized pp1ab	Reported cases by onset date	0.4949 (*p* < 0.0001)	5 days	Lara-Jacobo et al. (2022)

Oregon USA	6 WWTPs 22 pump stations 10,853 – 258,910	24h composite 1–3/week	Apr 2020 - May 2021	52	Pearson	log_10_ (viral conc.)	log_10_ (reported cases/10,000)	0.71 (*R*^2^ = 0.5)		Layton et al. (2022)
							log_10_ (estimated prevalence/10,000)	0.96 (*R*^2^ =0.91)		

North Carolina USA	19 WWTPs 3,500 – 550,000	24h composite 1–2/week	Jan 21 - Mar 22	1,783	Spearman	Arithmetic mean conc. of N1 & N2 genes normalized by flow rate and population	7-day rolling average of cases at the sewershed	0.47 – 0.88		Keshaviah et al. (2022)
							7-day rolling average of cases at the county	0.55 – 0.9		

Bratislava Slovak	2 WWTPs 0.6 million	24h composite weekly	Sep 2020 - Mar 2021	52	*R*^2^ of linear regression	$\sqrt{\text{total}\;\text{viral}\;\text{load}}$ (from both WWTPs)	$\sqrt{\text{daily}\;\text{positive}\;\text{tests}}$	0.5265	12 days	Krivoňáková et al. (2021)
						ln(total viral load) (from both WWTPs)	$\sqrt{\text{daily}\;\text{death}\;\text{cases}}$	0.6189	27 days	
							$\sqrt{\text{weekly}\;\text{positive}\;\text{test}}$	0.8378	2 weeks	
							$\sqrt{\text{weekly}\;\text{death}\;\text{cases}}$	0.8321	4 weeks	

Mendoza Argentina	2 WWTPs 1.2 million	Grab weekly/biweekly (11am-1pm)	Jul 2020 - Jan 2021		Pearson	Viral conc. by N1 gene	Weekly reported cases	0.3185 – 0.5468, (*p* ∈ [0.0069,0.1386])		Giraud-Billoud et al. (2021)
						Viral conc. by N2 gene		0.389 – 0.6282, (*p* ∈ [0.1514 – 0.3946])		

Nepal	2 WWTPs, river, hospital, sewer	Grab	Jul 2020 - Feb 2021	84	Pearson	Viral conc.	Weekly new cases	0.47 – 0.5 (*p* < 0.05)		Tandukar et al. (2022)

Porto Portugal	2 WWTPs 370,000	24h composite weekly	Sep 2020 - Mar 2021	81	*R*^2^ with linear regression	Viral conc. in liquid phase	7-day moving avg. of new cases	0.2935 – 0.4223 (*p* ∈ [4.6 × 10^−7^, 4.2 × 10^−5^])		Tomasino et al. (2021)
						Viral conc. in solid phase		0.1831 – 0.1865 (*p* = 1.4 × 10^−3^)		

Calgary Canada	3WWTPs 6 sewers 1,441,268	24h composite ~3/week at WWTP biweekly at sewers	Jun 2020 - May 2021	222 from wwtp, 192 from neighborhood	Pearson	Viral conc.	5-day rolling avg. of new cases	0.33 – 0.82 (*p* < 0.0001/*p* = 0.19)	−4 – 4 weeks	Acosta et al. (2022)

Ohio USA	9 WWTPs 14,000 – 900,000	24h composite 2/week	Jul 2020 - Jan 2021	250	Pearson	Viral conc.	Reported cases on sampling date	0.38 – 0.89		Ai et al. (2021)
						Viral conc.	Reported cases on sampling date	0.48 – 0.87		
					Spearman	Avg. viral conc. from all WWTPs	Reported cases on sampling date	0.7		
							3-day rolling avg. of reported cases	0.75		
							5-day rolling avg. of reported cases	0.77		
							7-day rolling avg. of reported cases	0.76		

Basel Switzerland	1 WWTP 273,075	24h composite during weekdays 48h composite during weekends 6/week	Jul 2021 - Dec 2021	179	Spearman	7-day median viral conc.	7-day median reported cases	0.9395	1 day	Bagutti et al. (2022)

Buenos Aires Argentina	3 WWTPs 1 manhole 3,500 – 35,407	Grab weekly (morning)	Jun 2020 - Apr 2021	172	Spearman	Viral conc.	10-day cumulative cases	0.476 – 0.795 (*p* ∈ [1.98 × 10^−10^, 0.001])		Barrios et al. (2021)
							15-day cumulative cases	0.443 – 0.807 (*p* ∈ [6.23 × 10^−11^, 0.003])		
							20-day cumulative cases	0.499 – 0.812 (*p* ∈ [4.085 × 10^−11^, 0.001])		

Scotland	28 WWTPs 4,128 – 605,569	24h composite weekly	May 2020 - Jan 2021	989	Spearman	Viral conc.	Reported cases in the previous week	0.79		Fitzgerald et al. (2021)
							Positive test rate	0.83		
						Viral load	Reported cases	0.91		
							Postive test rate	0.77		

Attica Greece	1 WWTP 3,652,013	Composite daily	Aug 2020 - Mar 2021	203	*R*^2^ of linear regression	Normalized viral load/100k inhabitants	4-day avg. positive cases	0.947	3 – 4 days	Galani et al. (2022)
							4-day avg. new hosp. admissions	0.888		
							4-day avg. new ICU admissions	0.877		

New York City USA	14WWTPs 120,000 – 1.2million	24h composite 1–2/week	Aug 2020 - Jan 2021	~770	Spearman	Viral load	7-day avg. new cases	0.38 – 0.81		Hoar et al. (2022)

Northern Nevada, USA	3 WWTPs 390,750	Grab 3–5/week, (9am-12pm) 24h composite in 1 WWTP 7/week (Jul 2021-Sept 2021)	Jun 2020 - Sep 2021	614	Spearman	Viral conc.	Reported cases on sampling date	0.353 – 0.615		Li et al. (2022)
						7-day avg. viral conc.	7-day avg. reported cases	0.472 – 0.79		
						Viral conc.	Lagged reported cases	0.232 – 0.635	7 days	
						7-day avg. viral conc.	Lagged 7-day avg. reported cases	0.415 – 0.793	7 days	

Hamilton USA	2 WWTPs 1 sewer 34,000 – 488,000	24h composite/3h composite weekly	May 2020 - Oct 2020	69	Pearson	Raw viral conc. by N1 gene	New cases	0.14 – 0.71		Nagarkar et al. (2022)
						Raw viral conc. by N2 gene		0.22 – 0.71		
						Raw viral load by N1 gene		−0.063 – 0.7		
						Raw viral load by N2 gene		0.014 – 0.7		
						Raw PMMoV normalized N1 gene		0.1 – 0.63		
						Raw PMMoV normalized N2 gene		0.15 – 0.61		
						OC43 recovery adjusted viral conc. by N1 gene		0.31 – 0.63		
						OC43 recovery adjusted viral conc. by N2 gene		0.31 – 0.68		
						OC43 recovery adjusted viral load by N1 gene		0.16 – 0.58		
						OC43 recovery adjusted viral load by N2 gene		0.17 – 0.64		
						OC43 recovery adjusted PMMoV normalized N1 gene		0.25 – 0.64		
						OC43 recovery adjusted PMMoV normalized N2 gene		0.28 – 0.7	

Utah USA	10 WWTPs 9,095 – 515,494	24h composite	Apr 2020 - May 2020	126	Spearman	Viral load/person/day	Daily new cases	0.54 (*p* < 0.001)		Weidhaas et al. (2021)
							Weekly cases/100k	0.82 – 0.96 (*p* < 0.003/*p* < 0.05)		
							Lagged weekly cases/100k	0.8 (*p* < 0.01)	1 week	

Dublin Ireland	1 WWTP 1.9 million	24h composite 2/week	Jun 2020 - Aug 2021	99	Spearman	First order difference in N1 viral load	First order difference in cases	0.5 (*p* < 0.001)	0	Reynolds et al. (2022)
						First order difference in N1 viral conc.		0.49 (*p* < 0.001)		

Catalonia Spain	10WWTPs 7,000 – 1.5million	24h composite	Mar 2020 - Nov 2020	185	*R*^2^ of linear regression	log(viral conc.)	log(cumulative cases in the following 7 days)	0.3839		Rusinol et al. (2021)
							log(cumulative cases in the previous 7 days)	0.2524		
						log(viral load)	log(cumulative cases in the following 7 days)	0.731		
							log(cumulative cases in the previous 7 days)	0.7092		
							log(cumulative cases in rolling 15 days)	0.6004		
			Jul 2020 - Nov 2020			log(viral conc.)	log(cumulative cases in the following 7 days)	0.2839		
							log(cumulative cases in the previous 7 days)	0.1354		
						log(viral load)	log(cumulative cases in the following 7 days)	0.7515		
							log(cumulative cases in the previous 7 days)	0.7293		
							log(cumulative cases in rolling 15 days)	0.6691		

Germany	9 WWTPs 120,000 – 2.4million	24h composite	Apr 2020		*R*^2^ of linear regression	Viral load	Cumulative cases	0.9730		Westhaus et al. (2021)
							Acute cases	0.9661		
							Creatinine corrected cumulative cases	0.9603		
							Creatinine corrected acute cases	0.9467		

Marseille France	Sewer 359,123 – 614,623	24h composite ~every 1.4days	Jul 2020 - Dec 2020	117	Cross correlation	Viral load	Reported cases	0.68 (*p* < 0.01)	0	Wurtz et al. (2021)

Frankfurt Germany	2 WWTPs 470,000 – 1.35 million	24h composite 2/week	Apr 2020 - Aug 2020	44	Spearman	Sum of viral load from both WWTPs	Reported cases/100k	0.7464 (*p* = 0.00217)		Agrawal et al. (2021)

Riyadh Saudi Arabia	3 WWTPs	Grab 1/month	Jun 2020 - Aug 2020	9	Spearman	*C_t_* by N1 gene	Hospital reported cases 2 week after sampling	0.42		Alahdal et al. (2023)
						*C_t_* by N2 gene		0.37		
						*C_t_* by E gene		0.42		

Ottawa Canada	1 WWTP 1million	24h composite, every 2 days	Jun 2020 - Aug 2020		Pearson	PMMoV normalized viral load with N1	Daily new cases	0.673 (*p* < 0.001)		D’Aoust et al. (2021a)
						PMMoV normalized viral load with N2 gene		0.648 (*p* < 0.001)		
						PMMoV normalized viral load with N1 gene	Positive test rate	0.468 (*p* < 0.001)		
						PMMoV normalized viral load with N2 gene		0.404 (*p* < 0.001)		
						PMMoV normalized viral load with N1 gene	Lagged daily new cases	0.703 (*p* < 0.001)	2 days	
						PMMoV normalized viral load with N2 gene		0.721 (*p* < 0.001)		
						PMMoV normalized viral load with N1 gene	Lagged hospital cases	0.741 (*p* < 0.001)	4 days	
						PMMoV normalized viral load with N2 gene		0.767 (*p* < 0.001)		

Ottawa & Gatineau Canada	2 WWTPs 280,000 – 1.1million	Grab every 2 weeks 24h composite every 2 days-1/week	Apr 2020 - Jun 2020		Pearson	Viral conc.	Daily cases	−0.209 – 0.399 (*p* < 0.00*/*p* = 0.003)		D’Aoust et al. (2021b)
							Active cases	−0.233 – 0.95 (*p* < 0.001/*p* = 0.003)		
							7-day rolling avg. positive rate	0.378 – 0.55 (*p* < 0.001/*p* = 0.003)		
						Viral load	Daily cases	−0.48 – 0.05 (*p* < 0.001/*p* = 0.01)		
							Active cases	−0.289 – 0.125 (*p* < 0.001/*p* = 0.298)		
							7-day rolling avg. positive rate	−0.274 – 0.178 (*p* ∈ [0.001,0.008])		
						PMMoV normalized viral load	Daily cases	−0.48 – 0.05 (*p* < 0.001/*p* = 0.01)		
							Active cases	−0.144 – 0.383 (*p* ∈ [0.003,0.049])		
							7-day rolling avg. positive rate	−0.022 – 0.639 (*p* ∈ [0.003,0.123])		

University of North Carolina Charlotte USA	building plumb & manholes	24h composite 3/week	Sep 2020 - Nov 2020	332	Pearson	Total number of positive wastewater samples	Daily new cases in the county	0.769		Gibas et al. (2021)

Bozeman USA	1 WWTP 49,831	24h composite	Mar 2020 - Apr 2020	17	Pearson	Viral conc.	Lagged cases by symptom onset date	0.972 – 0.995	−8 days	Nemudryi et al. (2020)
							Lagged positive tests	0.911 – 0.988	2 days	

Japan	2 WWTPs 1 manhole	Grab 1/week	Jun 2020 - Aug 2020	32	Spearman	Viral conc.	Number of case by report date	0.71 (*p* < 0.01)		Kitamura et al. (2021)
							Number of cases by symptom onset date	0.87 (*p* < 0.001)		

France	10 WWTPs 50,000 – 560,000	24h composite 2/week - 2/month	Jul 2020 - Dec 2020	138	Spearman	Viral conc. Viral load/100k inhabitant/day	log10 (7-day moving avg. incidence rate)	0.32 – 0.82		Lazuka et al. (2021)
								0.3 – 0.87		

Bangkok Tailand	19 WWTPs	24h composite	Jan 2021 - Apr 2021	132	Spearman	Positive rate of wastewater samples	Lagged 5-day avg. new cases	1	22 days	Sangsanont et al. (2022)
						log10(viral load)	Lagged new cases	0.85	23 – 24 days	

Cape Town South Africa	23 WWTPs	Grab 1/week (Monday)	Jul 2020 - Aug 2020	138	Spearman	Viral conc.	Reported cases	0.83 (*p* = 0.0416)		Street et al. (2021)

Stockholm Sweden	3 WWTPs 377,500 – 862,100	24h composite daily-1/week	Oct 2020 - Jan 2021 (Wave 2)	600	Pearson	PMMoV normalized total viral load from all WWTPs	Positive cases	0.84 (*p* < 0.0001)		Perez-Zabaleta et al. (2023)
							Number of patients in ICU	0.83 (*p* < 0.0001)		
							Number of death	0.88 (*p* < 0.0001)		
			Feb 2021 - May 2021 (Wave 3)			PMMoV normalized total viral load from all WWTPs	Positive cases	0.88 (*p* < 0.0001)		
							Number of patients in ICU	0.64 (*p* = 0.0008)		
			Apr 2020 - Jun 2022 (Entire Period)			PMMoV normalized total viral load from all WWTPs	Positive cases	0.86 (*p* < 0.0001)		
						Raw total viral load from all WWTPs		0.84 (*p* < 0.0001)		
						PMMoV normalized viral load	Positive cases in sewershed	0.85 – 0.88 (*p* < 0.0001)		

Valencia Spain	3 WWTPs 29,459	Grab 1/week (10am-11am)	Nov 2020 - May 2021 (Wave 3)	195	Pearson	Sum of viral conc. from all 3 WWTPs	14 day cumulative incidences	0.86	0	López-Peñalver et al. (2023)
							Positive cases	0.85	0	
							Hospital cases	0.76	0	
							Critical hospital cases	0.65	−3 days	
							Death cases	0.04		
			Jun 21 - Jul 21 (Wave 5)			Sum of viral conc. from all 3 WWTPs	14 day cumulative incidences	0.95		
							Positive cases	0.88		
							Hospital cases	0.64		
							Critical hospital cases	0.45		
							Death cases	0.29		
			Nov 21 - Jan 22 (Wave 6)			Sum of viral conc. from all 3 WWTPs	14 day cumulative incidences	0.79	−1 day	
							Positive cases	0.88	−2 days	
							Hospital cases	0.66	−1 day	
							Critical hospital cases	0.64	−1 day	
							Death cases	0.38		

Eastern upper Peninsula USA	13 WWTPs 3 sewers 280 – 19,668	Grab 1/week	Jun 2021 - Dec 2021		Spearman	Viral load	Clinical cases	0.48 – 0.89	0–7 days	Jarvie et al. (2023)

Curitiba Brazil	5 WWTPs 276,778 – 969,987	4h composite 8am - 12pm	Mar 2021 - Apr 2021 (Gamma wave)	458	Spearman	Viral load	Positive tests	0.819 (*p* < 0.01)	3 days	Belmonte-Lopes et al. (2023)
							Reported cases	0.786 (*p* < 0.01)	8 days	
							Active cases	0.747 (*p* < 0.01)	15 days	
			Apr 2021 - Nov 2021 (Delta wave)			Viral load	Positive tests	0.93 (*p* < 0.01)	3 days	
							Reported cases	0.938 (*p* < 0.01)	11 days	
							Active cases	0.936 (*p* < 0.01)	18 days	
			Nov 2021 - Nov 2022 (Omicron wave)			Viral load	Positive tests	0.927 (*p* < 0.01)	3 days	
							Reported cases	0.929 (*p* < 0.01)	5 days	
							Active cases	0.901 (*p* < 0.01)	10 days	

California USA	21 WWTPs 5 pump stations/manholes 2,200 – 4million	24h composite 1–5/week	Oct 2020 - Mar 2022	2,480	Kendall’s *τ*-b	Viral conc.	7-day moving avg. cases	0.57		Schill et al. (2023)
						Flow normalized viral conc.		0.58		
						PMMoV normalized viral conc.		0.47		

United Arab Emirates	50 WWTPs/pump stations, 150 schools/sewers, (453 locations)	24h composite	Aug 2020 - Jan 2021 (Wave 1)	16,858	Pearson	Positive rate of wastewater samples	Positive rate of clinical tests	0.7	0	Wadi et al. (2023)
			Jan 2021 - May 2021 (Wave 2)			Positive rate of wastewater samples	Positive rate of clinical tests	−0.768 (*p* < 0.0001)	0	
						Total viral load across the county	Weekly cases	−0.2 (*p* = 0.3999)		
			May 2021 - Dec 2021 (Wave 3)			Positive rate of wastewater samples	Positive rate of clinical tests	0.841 (*p* < 0.0001)	0	
						Total viral load across the county	Weekly cases	0.88 (*p* < 0.0001)		
			Dec 2021 - Apr 2022 (Wave 4)			Positive rate of wastewater samples	Positive rate of clinical tests	0.504 (*p* = 0.01)	0	
						Total viral load across the county	Weekly cases	0.671 (*p* = 0.001)		
			May 2020 - Jun 2022 (Entire Period)			Total viral load across the county	Weekly cases	0.334 (*p* = 0.004)		

Cape Town South Africa	Pump station at Cape Town, International Airport	Grab, 1/week (Monday)	Dec 2020 - Feb 2021 (Alert L3)	55	Spearman	log(viral conc.)	Reported cases in Cape Town	0.54 (*p* = 0.0084)		Nkambule et al. (2023)
			Oct 2021 - Dec 2021 (Alert L4)					0.69 (*p* = 0.0046)		
			Dec 2020 - Jan 2022					0.52 (*p* = 0.0001)	−1 week	

Michigan USA	2 WWTPs 25,000 – 110,267	24h composite, 1/week	Apr 2020 - Feb 2022	186	Pearson	Viral load/person/day with N1 gene	7-day avg. zipcode-level cases by symptom onset date	0.71 – 0.81 (*p* < 0.0001)		
							7-day avg. zipcode-level cases by referral date	0.62 – 0.72 (*p* < 0.0001)		Flood et al. (2023)
							7-day avg. county-level cases	0.53 – 0.59 (*p* < 0.0001)		
						Viral load/person/day with N2 gene	7-day avg. zipcode-level cases by symptom onset date	0.6 – 0.66 (*p* < 0.0001)		
							7-day avg. zipcode-level cases by referral date	0.51 – 0.6 (*p* < 0.0001)		
							7-day avg. county-level cases	0.46 – 0.56 (*p* < 0.0001)		

Northeastern Japan	2 WWTPs 200,000 – 500,000	Grab, 1/week-every 2 weeks	Aug 2020 - Nov 2021	81	Spearman	Viral conc.	New cases	0.61 (*p* < 0.0001)		Kitakawa et al. (2023)

Shenzhen China	2 WWTPs from hospital (emergency quarantine & whole hospital)	Grab 3/day (8am, 1pm, 6pm)	Aug 2022 - Sept 2022		Pearson	Emergency area viral conc.	Reported cases	0.76 (*p* = 2.9 × 10^−6^)		Ou et al. (2023)
							New cases	0.57 (*p* = 1.6 × 10^−3^)		
						Emergency area 10-day avg. viral conc.	Reported cases	0.99 (*p* = 1 × 10^−13^)		
							New cases	0.99 (*p* = 8.4 × 10^−12^)		
						Whole hospital viral conc.	Reported cases	0.64 (*p* = 2.7 × 10^−4^)		

National University of Singapore	7 sites divided into 28 discharge chambers 9,090	6h composite/12h composite	Jan 2021 - Mar 2022		Spearman	Aggregated weekly viral conc. at each site	Weekly reported cases at each site	0.5 – 0.76 (*p* < 0.05)	2–9 days	Mohapatra et al. (2023)
						aggregated weekly viral conc. over the campus	Weekly reported cases	0.76 (*p* < 0.05)		
						PMMoV normalized viral conc.		0.78 (*p* < 0.05)		

**Table 2:** Current Wastewater Datasets for COVID-19 Surveillance.

Country	Data Granularity	Area Covered	Time Granularity	Time Span	Live	Websites
USA	Country/Region/County	Nationwide (240 counties)	Weekly	Jan 2020 - Current	Yes	https://biobot.io/data/
USA	County/State/WWTP	Nationwide	Weekly	Aug 2022 - Current	Yes	https://covid.cdc.gov/covid-data-tracker/#wastewater-surveillance
USA	Country/Region/WWTP	Nationwide	>1/week	Jul 2020 - Current	Yes	https://data.wastewaterscan.org/
USA	WWTP	Gilbert, AZ	>1/week	Jun 2020 - May 2022	No	https://tog.maps.arcgis.com/apps/dashboards
USA	WWTP	Flagstaff, AZ	Weekly	Oct 2021 - Current	Yes	https://pathogen-intelligence.tgen.org/VECTRSurveillance/flagstaff/
USA	Community	Tempe, AZ	Weekly	Mar 2020 - Current	Yes	https://wastewater.tempe.gov/pages/biomarker-covid19#COVID-19-Dashboard
USA	WWTP	California	>1/week	Dec 2020 - Current	Yes	https://www.cdph.ca.gov/Programs/CID/DCDC/Pages/COVID-19/CalSuWers-Dashboard.aspx
USA	WWTP	Davis, CA	Daily	Nov 2020 - Current	Yes	https://healthydavistogether.org/wastewater/
USA	Community	UC Davis, CA	Daily	Sept 2021 - Current	Yes	https://campusready.ucdavis.edu/testing-response/dashboard
USA	Community	Marin, CA	1–3/week	Sept 2021 - Current	Yes	https://coronavirus.marinhhs.org/surveillance
USA	County/WWTP/Community	Central valley, CA	3/week	Nov 2020 - Current	Yes	https://healthycvtogether.org/data-main/covid/
USA	WWTP	Santa Clara, CA	Daily	Oct 2020 - Current	Yes	https://covid19.sccgov.org/dashboard-wastewater#3925188384-738865471
USA	WWTP	San Luis Obispo, CA	Daily	May 2022 - Current	Yes	https://www.slocounty.ca.gov/COVID-19/Data.aspx#Wastewater
USA	WWTP	Santa Barbara, CA	Weekly	May 2021 - Current	Yes	https://www.sbcwastewater.org/dashboard
USA	WWTP	San Bernardino, CA	Weekly	Sept 2020 - May 2023	No	https://lookerstudio.google.com/u/0/reporting/430e67c8-acaf-4574-a2d4-48d0b665ab05/page/jMhOC
USA	WWTP	Palm Springs, CA	Weekly	Jan 2022 - Jul 2023	No	https://www.palmspringsca.gov/government/departments/community-economic-development-department/wastewater-treatment-plant-covid-19-test-reports
USA	WWTP	UC San Diego, CA	Daily	Feb 2021 - Current	Yes	https://blink.ucsd.edu/safety/resources/public-health/covid-19/dashboard.html
USA	WWTP	San Diego, CA	Daily	Feb 2021 - Current	Yes	https://searchcovid.info/dashboards/wastewater-surveillance/
USA	WWTP	Colorado	>1/week	Aug 2021 - Current	Yes	https://cdphe.maps.arcgis.com/apps/dashboards/d79cf93c3938470ca4bcc4823328946b
USA	WWTP	4 cities in CT	Daily	Aug 2022 - Current	Yes	https://yalecovidwastewater.com/sars-cov-2/
USA	WWTP/Building	UConn, CT	>1/week	Apr 2023 - Current	Yes	https://covid-testing.uconn.edu/dashboard/
USA	WWTP/Community	New Castle, DE	Weekly	May 2020 - Sept 2023	No	https://techimpact.shinyapps.io/ncco_wastewater/
USA	WWTP	St. Augustine, FL	Weekly	Apr 2020 - Mar 2021	No	https://data-staug.opendata.arcgis.com/apps/STAUG::covid-19-wastewater-testing-dashboard/explore
USA	County/WWTP	Georgia	2/week	Apr 2022 - Current	Yes	https://wastewatersurveillance.s3.amazonaws.com/ExternalNWSS_20231011.html#summary-report
USA	Community/Building	Georgia Tech, GA	>1/week	Aug 2022 - Current	Yes	https://health.gatech.edu/coronavirus/monitoring-covid/
USA	County	Hawaii	Biweekly	Sept 2022 - Current	Yes	https://health.hawaii.gov/coronavirusdisease2019/what-you-should-know/covid-19-data-reports/
USA	City	Boise, ID	Daily-3/week	May 2020 - Current	Yes	https://www.cityofboise.org/departments/mayor/covid-19-information/wastewater-testing/
USA	County/WWTP	Idaho	>1/week	Jun 2021 - Current	Yes	https://public.tableau.com/app/profile/idaho.division.of.public.health/viz/DPHIdahoCOVID-19Dashboard/Home
USA	WWTP	Illinois	>1/week	Nov 2021 - Current	Yes	https://iwss.uillinois.edu/wastewater-treatment-plants/?page=1
USA	WWTP	Kendall, IL	>1/week	Nov 2020 - Jun 2022	No	https://portal.rjngroup.com/arcgisportal/apps/opsdashboard/index.html#/594d4b1b2dd840958cedb50b1381982b
USA	WWTP/Community	Chicago	2/week	Mar 2022 - Current	Yes	https://www.chicago.gov/city/en/sites/covid-19/home/covid-19-wastewater-surveillance.html
USA	State/WWTP	Indiana	>1/week	Feb 2020 - Current	Yes	https://www.coronavirus.in.gov/indiana-covid-19-dashboard-and-map/wastewater-dashboard/
USA	WWTP	Cedar Rapids, IA	>1/week	Oct 2021 - Current	Yes	https://www.cedar-rapids.org/residents/utilities/covid-19.php
USA	City	Louisville, KY	Weekly	Sept 2022 - Current	Yes	https://louisville.edu/envirome/thecoimmunityproject/dashboard
USA	WWTP	Maine	Weekly	May 2020 - Aug 2023	No	https://www.maine.gov/dhhs/mecdc/infectious-disease/epi/airborne/coronavirus/wastewater-reports.shtml
USA	WWTP/Community	Montgomery, MD	2/week	Oct 2022 - Current	Yes	https://montgomerycountymd.gov/covid19/data/wastewater-surveillance.html
USA	WWTP/Community	Eastern MA	Daily	Mar 2020 - Current	Yes	https://www.mwra.com/biobot/biobotdata.htm
USA	Community	Boston, MA	2/week	Jan 2023 - Current	Yes	https://www.boston.gov/government/cabinets/boston-public-health-commission/covid-19-boston#wastewater-reports
USA	WWTP	Massachusetts	>1/week	Feb 2020 - Current	Yes	https://www.mass.gov/info-details/wastewater-surveillance-reporting
USA	Community	Cambridge	Weekly	Oct 2020 - Current	Yes	https://cityofcambridge.shinyapps.io/COVID19/#shiny-tab-wastewater
USA	WWTP	Southeast Michigan	Daily	Jul 2021 - Current	Yes	https://um.wastewatermonitoring.dataepi.org/
USA	WWTP	Michigan	Weekly	Jun 2021 - Current	Yes	https://www.michigan.gov/coronavirus/stats/wastewater-surveillance/dashboard/sentinel-wastewater-epidemiology-evaluation-project-sweep
USA	WWTP/Community	Michigan	>1/week	Apr 2020 - Dec 2020	No	https://storymaps.arcgis.com/stories/f2996168197c4bbfa05e76b893fd9a8e
USA	WWTP/Community/Building	Michigan	Weekly	Apr 2020 - Current	Yes	https://gisportal.state.mi.us/portal/apps/insights/index.html#/view/52bbb104ed574887918f990af9f3debe
USA	WWTP	Twin Cities, MN	Daily	Nov 2020 - Aug 2023	No	https://metrotransitmn.shinyapps.io/metc-wastewater-covid-monitor/
USA	State/WWTP	Minnesota	>1/week	Jan 2023 - Current	Yes	https://umn.maps.arcgis.com/apps/dashboards/fd0350c812334c5f9733ca5b6186db0d
USA	State/WWTP	Missouri	Weekly	Jul 2020 - Current	Yes	https://storymaps.arcgis.com/stories/f7f5492486114da6b5d6fdc07f81aacf
USA	City	Montana	>1/week	Mar 2020 - Dec 2022	No	https://www.healthygallatin.org/coronavirus-covid-19/wastewater-data/
USA	WWTP	Grand Island, NE	Weekly	Feb 2022 - Current	Yes	https://cdhd.ne.gov/how-do-i/find/covid-wastewater-reports.html
USA	City/Community/Building	Southern Nevada	Weekly/Monthly	Aug 2020 - Aug 2023	Yes	https://empower.unlv.edu/
USA	County/City	Nevada	Weekly	May 2020 - Oct 2022	No	https://thenevadaindependent.com/coronavirus-data-nevada
USA	WWTP	New Hampshire	Weekly	Oct 2022 - Current	Yes	https://wisdom.dhhs.nh.gov/wisdom/dashboard.html?topic=covid-19&subtopic=recurring-updates&indicator=covid-19-wastewater#tabnavbarid
USA	WWTP	New York	>1/week	Aug 2020 - Current	Yes	https://mbcolli.shinyapps.io/SARS2EWSP/#
USA	WWTP	North Carolina	>1/week	Jan 2021 - Current	Yes	https://covid19.ncdhhs.gov/dashboard/wastewater-monitoring
USA	WWTP	Western NC	>1/week	Jan 2021 - Jul 2022	No	https://wastewater.covid19.mathematica.org/
USA	WWTP	Huron, OH	2/week	Aug 2023 - Oct 2023	No	https://www.huroncohealth.com/public-information
USA	WWTP	Ohio	>1/week	Aug 2020 - Sept 2023	No	https://www.neorsd.org/ohio-coronavirus-wastewater-monitoring-network-data-for-northeast-ohio-regional-sewer-district/
USA	State/County/City	Oklahoma	Weekly	Jul 2021 - Current	Yes	https://www.tulsa-health.org/community-health/illness-disease/coronavirus-disease-2019-covid-19/tulsa-county-covid-19-data
USA	City	Oregon	>1/week	Sept 2020 - Current	Yes	https://public.tableau.com/app/profile/oregon.health.authority.covid.19/viz/OregonsSARS-CoV-2WastewaterMonitoring/WastewaterDashboard
USA	County/WWTP	Pennsylvania	>1/week	Jul 2022 - Current	Yes	https://www.arcgis.com/apps/dashboards/ee27ed0a3a9f4ddbb380ec0eb1369a84
USA	County	Indiana, PA	Weekly	Aug 2020 - Sept 2023	No	https://www.indianaboro.com/news/categories/wastewater-surveillance
USA	County	Allegheny, PA	>1/week	Nov 2021 - Current	Yes	https://mcba.autonlab.org/covidashboard/public
USA	County	Chattanooga, TN	Weekly	May 2020 - Mar 2023	No	https://connect.chattanooga.gov/covid-biobot-analysis-reports/
USA	WWTP	Houston, TX	Weekly	Jul 2020 - Current	Yes	https://covidwwtp.spatialstudieslab.org/?data_id=dataSource_14-b3436880c1ff47efb60e44ac78851c5e%3A20055%2CdataSource_18-WWTP_List%3A6&page=page_0
USA	WWTP	Utah	>1/week	Mar 2020 - Current	Yes	https://udwq.shinyapps.io/sarscov2_surv/
USA	WWTP	Burlington, VT	Weekly	Jul 2022 - Current	Yes	https://www.burlingtonvt.gov/covid-19/wastewater
USA	WWTP	Virginia	>1/week	Sept 2021 - Current	Yes	https://www.vdh.virginia.gov/coronavirus/see-the-numbers/covid-19-data-insights/sars-cov-2-in-wastewater/
USA	WWTP	Spokane, WA	Weekly	Dec 2021 - Current	Yes	https://covid.srhd.org/topics/spokane-county-case-data
USA	WWTP	Washington	>1/week	Oct 2021 - Current	Yes	https://doh.wa.gov/data-and-statistical-reports/diseases-and-chronic-conditions/communicable-disease-surveillance-data/respiratory-illness-data-dashboard#WasteWater
USA	State/WWTP	West Virginia	Weekly	Oct 2022 - Current	Yes	https://wvuvectors.shinyapps.io/WaICH-WV/
USA	State/WWTP	Wisconsin	>1/week	Sept 2020 - Current	Yes	https://www.dhs.wisconsin.gov/covid-19/wastewater.htm
USA	WWTP	Wyoming	>1/week	Oct 2020 - Dec 2021	No	https://covidwastewatermonitor.wyo.gov/
Canada	Region/WWTP	Nationwide	>1/week	Oct 2020 - Current	Yes	https://health-infobase.canada.ca/covid-19/wastewater/
Canada	WWTP	Eastern Ontario	>1/week	Aug 2023 - Current	Yes	https://eohu.ca/en/covid/covid-19-status-update-for-eohu-region
Canada	WWTP	Kingston, ON	3–5/week	Nov 2022 - Current	Yes	https://app.powerbi.com/view?r=eyJrIjoiMzg5ZGFmNTAtZDcxNC00N2NiLTg0YmUtMGY2ZmM5ODZkOTVjIiwidCI6Ijk4M2JmOTVjLTAyNDYtNDg5My05MmI4LTgwMWJkNTEwYjRmYSJ9&pageName=ReportSection56a7adbd9a3e98e0661d
Canada	WWTP	Windsor-Essex, ON	5/week	Dec 2020 - Current	Yes	https://www.wechu.org/reports/local-covid-19-surveillance
Canada	WWTP	London, ON	3–5/week	Oct 2021 - Current	Yes	http://www.519covid.ca/
Canada	WWTP	Waterloo, ON	>1/week	Jan 2021 - Apr 2023	Yes	https://www.regionofwaterloo.ca/en/health-and-wellness/covid-19-wastewater-surveillance.aspx#
Canada	Region/City	Wellington, Dufferin, and Guelph, ON	Daily	Sept 2021 - Current	Yes	https://bi.wdgpublichealth.ca/respiratory-dashboard/
Canada	WWTP	Halton, ON	3–5/week	Jan 2023 - Current	Yes	https://www.halton.ca/For-Residents/Immunizations-Preventable-Disease/Diseases-Infections/COVID-19
Canada	WWTP	Toronto	>1/week	Feb 2021 - Current	Yes	https://www.toronto.ca/community-people/health-wellness-care/health-programs-advice/respiratory-viruses/covid-19/covid-19-pandemic-data/covid-19-wastewater-surveillance/
Canada	Province/Region	Ontario	3–5/week	Oct 2022 - Current	Yes	https://www.publichealthontario.ca/en/Data-and-Analysis/Infectious-Disease/COVID-19-Data-Surveillance/Wastewater
Canada	WWTP	York, ON	Daily	Nov 2020 - Current	Yes	https://www.york.ca/health/covid-19/covid-19-york-region#.Yd3nnYjMK3A?utm_source=newmarkettoday.ca&utm_campaign=newmarkettoday.ca%3A%20outbound&utm_medium=referral
Canada	WWTP	Peterborough, ON	5/week	Jul 2022 - Current	Yes	https://app.powerbi.com/view?r=eyJrIjoiMDRhYWQ1NzktNjlkMi00YTQ2LWI0NDItOTQ0ZDU2MDk3YTllIiwidCI6IjQ4OTJlODVlLTM1NzEtNGUzNy1hZjU1LTE4NTU3MjA2NDBjOCJ9&pageName=ReportSectionb42f1cb240c9ad8780d8
Canada	WWTP	Greater Sudbury, ON	3–5/week	Jan 2021 - Current	Yes	https://www.greatersudbury.ca/live/covid-19-coronavirus/measuring-sars-cov-2-in-wastewater-covid-19/
Canada	WWTP	Thunder Bay, ON	3–5/week	Dec 2021 - Current	Yes	https://www.tbdhu.com/datadashboard
Canada	City	Alberta	>1/week	Jul 2023 - Current	Yes	https://covid-tracker.chi-csm.ca/
Canada	WWTP	British Columbia	2–3/week	Jan 2022 - Current	Yes	https://bccdc.shinyapps.io/respiratory_wastewater/#Viral_Load_Summary
Brazil	City	6 cities	Daily	Jun 2021 - Mar 2022	No	https://app.powerbi.com/view?r=eyJrIjoiNzMxYjdiZGYtZDVjNy00NTMwLWIwZmItYmQwOWJhNzk3YmU1IiwidCI6Ijc1NmU3MTc4LTA1ZmYtNGVmYy05OTY2LTU2ODFlNjE2MjA3MCJ9&pageName=ReportSectiond497bb36400a320db4c7
Brazil	City	2 cities	Monthly	Apr 2020 - Aug 2022	No	https://coronavirus.saude.mg.gov.br/transparencia/monitoramento-covid-esgotos
New Zealand	Region/City	16 regions	Weekly	Jul 2022 - Current	Yes	https://esr-cri.shinyapps.io/wastewater/#region=Wellington&log_or_linear=linear&period=twelveMonthsButton
Australia	WWTP	Perth	Weekly	Jul 2022 - Current	Yes	https://www.health.wa.gov.au/articles/a_e/coronavirus/covid19-wastewater-surveillance
Australia	Region/City	Queesland	Daily	Jul 2020 - Sept 2022	No	https://www.data.qld.gov.au/dataset/queensland-wastewater-surveillance-for-sars-cov-2
Australia	Region/City	Sydney	Biweekly	Feb 2022 - Current	Yes	https://www.health.nsw.gov.au/Infectious/covid-19/Pages/reports.aspx
South Africa	Province/District/WWTP	9 Province/16 District/85 WWTP	Weekly	Feb 2021 - Dec 2022	No	https://wastewater.nicd.ac.za/
South Africa	Province	5 Province/10 District/76 WWTP	Weekly	Nov 2021 - Apr 2023	No	https://www.samrc.ac.za/wbe/
Austria	WWTP	10 regions in Austria	Daily	Jan 2022 - Current	Yes	https://abwassermonitoring.at/dashboard/
Belgium	WWTP/Regions	206 Municipalities in Belgium	2/week	Sep 2020 - Current	Yes	https://wastewater.sciensano.be/dashboard/covid19/en/
Czech Republic	WWTP	4 regions in Czech Republic	Weekly	Apr 2020 - Jan 2023	No	https://heis.vuv.cz/data/webmap/datovesady/projekty/covmon/default.asp?lang=cs&tab=6&wmap=
Cyprus	WWTP	Cyprus	Weekly	Oct 2021 - Oct 2022	No	https://covid-pulse.cy/
Denmark	Region	5 regions in Denmark	Weekly	Aug 2022 - Current	Yes	https://www.ssi.dk/sygdomme-beredskab-og-forskning/sygdomsovervaagning/c/covid-19—spildevandsovervaagning
Germany	WWTP/City	Berlin	Weekly	Apr 2022 - Current	Yes	https://data.lageso.de/lageso/corona/corona.html#abwasser
Germany	Region	Bavarian municipalitie	Weekly	Nov 2022 - Current	Yes	https://www.bay-voc.lmu.de/abwassermonitoring
Greece	City	Athens	Daily	Apr 2020 - Nov 2022	No	http://trams.chem.uoa.gr/covid-19/
Filand	City	13 Cities/Regions in Finland	Weekly/Monthly	Oct 2022 - Current	Yes	https://www.thl.fi/episeuranta/jatevesi/wastewater_weekly_report.html
Lithuania	WWTP/City	3 regions in Lithuania	Weekly	Nov 2022 - Current	Yes	https://nvsc.lrv.lt/lt/informacija-visuomenei-apie-covid-19/covid/sars-cov-2-stebejimas-nuotekose/stebesenos-rezultatai
Luxembourg	WWTP	Luxembourg	Weekly	May 2020 - Current	Yes	https://www.list.lu/en/covid-19/coronastep/
Netherlands	Regions	Netherlands	3/week	Sep 2020 - Current	Yes	https://coronadashboard.riiksoverheid.nl/landeliik/rioolwater
Norway	WWTP/City	5 areas in Norway	Weekly	May 2022 - Current	Yes	https://www.fhi.no/en/in/surveillance/wastewater-surveillance-of-infectious-diseases/results-from-wastewater-surveillance/
Poland	WWTP	Warsaw	Weekly	Mar 2022 - Current	Yes	https://www.mpwik.com.pl/view/monitoring-wirusa-sars-cov-2-w-sciekach-w-aglomeracji-warszawskiej#S.embed_link-K.C-B.1-L.4.zw
Slovakia	Region	7 regions in Slovakia	Weekly	May 2021 - Current	Yes	https://www.uvzsr.sk/sk/web/uvzen
Slovenia	Country	12 regions in Slovenia	Weekly	Mar 2020 - Current	Yes	https://covid-19.sledilnik.org/en/stats
Spain	Region	5 regions in Spain	Weekly	Apr 2020 - Mar 2022	No	https://edarbens.es/covid19/
Spain	Region	Catalonia	Weekly	Jun 2020 - Current	Yes	https://sarsaigua.icra.cat/
Spain	Region	Madrid	Weekly	Feb 2021 - Current	Yes	https://www.canaldeisabelsegunda.es/sistema-vigia
Sweden	WWTP/City	26 WWTPs in Sweden	Weekly/Monthly	Feb 2020 - Current	Yes	https://www.pathogens.se/dashboards/wastewater/
Switzerland	WWTP/City	Switzerland	Daily/Weekly	Mar 2020 - Current	Yes	https://sensors-eawag.ch/sars/lugano.html
Switzerland & Lichtenstein	WWTP/City	Switzerland & Lichtenstein	Daily/Weekly	Feb 2022 - Current	Yes	https://www.covid19.admin.ch/en/epidemiologic/waste-water
Switzerland	WWTP/City	14 Regions in Switzerland	Daily/Weekly	Jan 2020 - Current	Yes	https://cov-spectrum.org/story/wastewater-in-switzerland
United Kingdom	Region	Scotland	Daily/Weekly	Jun 2020 - Current	Yes	https://informatics.sepa.org.uk/RNAmonitoring/
United Kingdom	Region	Scotland	Daily/Weekly	May 2020 - Current	Yes	https://scotland.shinyapps.io/phs-respiratory-covid-19/
United Kingdom	Region	England	Weekly	Jul 2021 - Mar 2022	No	https://www.gov.uk/government/collections/monthly-statistics-for-the-environmental-monitoring-for-health-protection-emhp-wastewater-program-england#latest-report
United Kingdom	Region	Wales	5/week	Feb 2022 - Jul 2023	No	https://www.gov.wales/wastewater-monitoring-reports-coronavirus
Bangladesh	Region	6 regions in Bangladesh		Nov 2021 - Aug 2023	No	https://erin-wettstone.shinyapps.io/Dashboard_V6/
China	Region	Hongkong	Weekly	May 2022 - Current	Yes	https://www.chp.gov.hk/en/resources/29/100148.html
India	WWTP/City	Bangalore	Weekly	Aug 2021 - Jun 2023	No	https://storymaps.arcgis.com/stories/c42be68c85634d19a5d92873a10bda66
India	City	Jodhpur	4/week	Feb 2023 - Jun 2023	No	https://storymaps.arcgis.com/stories/d376ct3e75204234a9dc6541ecad5a98
India	City	Pune	Daily/Weekly	Aug 2021 - Current	Yes	https://www.pkc.org.in/pkc-focus-area/health/waste-water-surveillance/wws-covid-dashboard-pune/
Israel	Region	Israel	Weekly	May 2022 - Jul 2023	No	https://app.powerbi.com/view?r=eyJrIjoiOTYwNDQ3NzItMTk5Ni00NzNmLThhMmEtMzk3NmI1NmFkZjhjIiwidCI6ImIzYzdlZDM0LWQxZjAtNDg5Zi05YzllLWE0YmNlYTk0YmJlNCIsImMiOjl9&pageName=ReportSection0f78f45a748a997ecd43
Japan	Region	Komatsu City	Weekly	Dec 2022 - Current	Yes	https://www.city.komatsu.lg.jp/soshiki/jougesuidoukanri/surveillance/14588.html

## Footnotes

1 In this paper, our primary focus is on the surveillance perspective of WBE when using the term ‘WBE’.

2 CrI: Credible Interval.
